# Urinary sediment miRNAs reflect tubulointerstitial damage and therapeutic response in IgA nephropathy

**DOI:** 10.1186/s12882-017-0482-0

**Published:** 2017-02-15

**Authors:** Shuang Liang, Guang-Yan Cai, Zhi-Yu Duan, Shu-wen Liu, Jie Wu, Yang Lv, Kai Hou, Zuo-xiang Li, Xue-Guang Zhang, Xiang-Mei Chen

**Affiliations:** Department of Nephrology, Chinese PLA General Hospital, Chinese PLA Institute of Nephrology, State Key Laboratory of Kidney Diseases, National Clinical Research Center for Kidney Diseases, 28 Fuxing Road, Beijing, 100853 China

**Keywords:** IgA nephropathy, Urinary sediment, miRNAs, Tubulointerstitial damage, Therapeutic response

## Abstract

**Background:**

Immunoglobulin A nephropathy (IgAN) is the most common glomerulonephritis worldwide. The clinical spectrum of IgAN varies from minor urinary abnormalities to rapidly progressive renal failure. Evaluation of the disease by repeated renal biopsy is not practical due to its invasive procedure. Urinary sediment miRNAs promise to serve as non-invasive biomarkers to assess kidney injury of IgAN.

**Methods:**

Fifty two biopsy-proven IgAN patients and twenty five healthy controls were enrolled in the study. Urinary sediment miRNAs were extracted. Expressions of miR-34a, miR-205, miR-21, miR-146a and miR-155 were quantified by real-time quantitative polymerase chain reaction (RT-QPCR). The receiver operating characteristic (ROC) curve was used to investigate the value of the miRNAs for predicting diagnosis of IgAN and evaluating histopathological injury. The patients were treated according to the Kidney Disease: Improving Global Outcomes (KDIGO) guidelines and followed up. The roles of miRNAs in reflecting therapeutic efficacy and disease progression were analyzed.

**Results:**

1. The IgAN group had significantly lower urinary miR-34a, miR-205, and miR-155, but higher miR-21 levels than controls. The ROC revealed that urinary miR-34a ≤ 0.047, miR-205 ≤ 0.209, miR-21 ≥ 0.461 and miR-155 ≤ 0.002 could distinguish patients with IgAN from healthy ones. In addition, miR-205 ≤ 0.125 and miR-21 ≥ 0.891 can distinguish IgAN patients with severe tubular atrophy/interstitial fibrosis from those with mild tubular atrophy/interstitial fibrosis. 2. After a mean 15.19 months follow-up, the reduction of proteinuria (g/24 h/year) was positively correlated with baseline urinary miR-21 and inversely correlated with miR-205. The levels of baseline eGFR and miR-205 in the complete remission group were significantly higher than non-complete remission group (*p* < 0.001; *p* = 0.018), while proteinuria, miR-21 and miR-146a were lower than non-complete remission group (*p* = 0.002; *p* = 0.021; *p* = 0.009). But multivariate analysis revealed that only baseline eGFR correlated with the remission of IgAN (*p* = 0.001, OR = 1.042).

**Conclusions:**

The levels of some urinary sediment miRNAs, especially baseline miR-21 and miR-205, may be used as potential prognostic markers for evaluating the tubulointerstitial damage of IgAN. Furthermore, baseline levels of urinary miRNAs may be predictors of therapeutic efficacy and disease progression.

**Electronic supplementary material:**

The online version of this article (doi:10.1186/s12882-017-0482-0) contains supplementary material, which is available to authorized users.

## Background

Immunoglobulin A nephropathy (IgAN) is the most common form of primary glomerulonephritis throughout the world, characterized by deposition of polymeric IgA in the mesangium, mesangial proliferation, and activation of proinflammatory and profibrotic mediators [1–3]. About 15–40% of patients with IgAN will progress to end-stage renal disease (ESRD) in 5–25 years [4]. Repeated renal biopsy is not practical to evaluate the disease severity and progression because it is an invasive technique. Thus, the investigation of non-invasive novel biomarkers based on the pathogenesis of IgAN has been a research focus.

MicroRNAs (miRNAs) are noncoding, single-stranded RNA molecules, regulating gene expression through post-transcriptional degradation of messenger RNA or translational inhibition of protein synthesis [5]. Dysregulation of miRNAs has been associated with many human diseases.

Recently, it has been reported that miR-34a is involved in cell proliferation. Down-regulation of miR-34a has been shown in different types of cancer, such as pancreatic cancer [6] and colon cancer [7]. Moreover, miR-34a declines the proliferation activity of mesangial cells in the anti-Thy1 mesangial proliferative glomerulonephritis rat models [8]. IgA nephropathy is characterized by mesangial cell proliferation and increased matrix. Thus, we hypothesized that miR-34 might participate in the pathological process of mesangial cell proliferation of IgAN.

Previous data reported that miR-205 inhibited cell proliferation and induced apoptosis in melanoma cells, through downregulated E2F1, a critical factor involved in cell cycle progression from G1 to S phase [9]. In addition, epithelial-mesenchymal transition (EMT) plays an important role in tumor invasion and metastasis. Studies have indicated that miR-205 is a negative regulator of EMT, by repressing ZEB1 and ZEB2, major transcription repressors of E-cadherin, a key marker of epithelial cells [10, 11]. We speculated that miR-205 might be involved in the process of cell proliferation and/or EMT of IgAN.

Recent studies reported a major role of miR-21 in mediating the pathogenic activation of lung and cardiac fibroblasts and, ultimately, fibrosis [12, 13]. An abundance of miR-21 is also observed in both tubulointerstitial and glomerular area where fibrosis happens in mouse models of obstructive and diabetic nephropathy [14, 15]. Transforming growth factor beta (TGF-β) is a well-known mediator of renal fibrosis [16], and miR-21 is positively induced by TGF-β signaling [17]. Given the established role of miR-21 in tissue fibrosis, we decided to assess whether urinary miR-21 was a reliable noninvasive marker of kidney fibrosis in IgAN.

Previous studies have proven that miR-146a and miR-155 are two important regulators in both immune and inflammatory response [18, 19]. For instance, expressions of miR-146a and miR-155 were higher in synovial fibroblasts of rheumatoid arthritis (RA) patients as compared with controls [20, 21]. MiR-146a expression was elevated in the tissues associated with chronic inflammatory diseases such as psoriasis [22]. An up-regulation of miR-155 was also observed after stimulation in vitro with TNF-α, IL-1β and toll-like receptor (TLR) ligand. Therefore, we hypothesized that miR-146a and miR-155 might be involved in the pathological process of immune and inflammatory response of IgAN.

In this study, we evaluated the levels of miR-34a, miR-205, miR-21, miR-146a, and miR-155 in the urine sediments of IgAN patients, aiming to explore the roles of urinary miRNA levels in diagnosing and predicting the progression of IgAN.

## Methods

### Enrollment of subjects

A total of 52 patients with biopsy-proven IgAN, and 25 healthy normal control (NC) participants matched by age and sex were enrolled in the study. The inclusion criteria were: patients signed informed consent; age ≥ 18. We excluded the following: patients with secondary IgA nephropathy; patients who had received corticosteroids or immunosuppressants before the beginning of this study; patients who had undergone kidney transplantation and who were undergoing dialysis; patients with systemic diseases such as diabetes; the number of glomerulus in renal biopsy tissues ≤8; patients who were pregnant, planning a pregnancy, or those who were breastfeeding. After the renal biopsy, the patients were treated with corticosteroids or angiotensin-converting enzyme inhibitors (ACEIs)/angiotensin receptor blockers (ARBs), according to the Kidney Disease: Improving Global Outcomes (KDIGO) guidelines and followed up for at least 12 months. Complete remission (CR) was defined as the absence of proteinuria (proteinuria < 0.3 g/24 h) along with the lack of worsening of renal function. Partial remission (PR) was defined as a ≥50% reduction in proteinuria from baseline. No response (NR) was defined as a <50% reduction in proteinuria [23, 24]. Glomerular filtration rate (GFR) was estimated by the chronic kidney disease epidemiology (CKD-EPI) equation [25]. The study was approved by the Clinical Research Ethical Committee of Chinese PLA General Hospital.

### Sample preparation

Morning urine specimens of 150–250 ml from patients before renal biopsy and from controls were collected and processed immediately or stored at 4 °C overnight. Then, the urine samples were centrifuged at 3000 g for 30 min and at 13,000 g for 5 min at 4 °C. The supernatants were discarded, and the urinary sediments were then stored at −80 °C until use. The kidney tissues were obtained by method of percutaneous renal biopsy. Sections were stained routinely by hematoxylin and eosin (H & E), periodic acid–Schiff stain (PAS) and periodic acid silver methenamine (PASM).

### Measurement of miRNA levels

Total RNA was extracted from urinary sediments using Trizol® (Invitrogen) according to the manufacturer’s instructions. Briefly, the tube containing the urinary sediments, Trizol, and chloroform was centrifuged. Subsequently, the remaining aqueous phase supernatant was removed into another tube, and equal volume of isopropanol was added, followed by mixing and incubation in ice. Then, samples were centrifuged and the supernatant discarded. RNA pellets were washed two times with 75% ethanol in diethylpyrocarbonate (DEPC) and then dried at room temperature, and DEPC was added. The RNA was used immediately or stored at −80 °C in 75% alcohol.

Micute miRNA First-Strand cDNA Synthesis Kit (TIANGEN, CHINA) was used for reverse transcription. For Poly(A), total RNA was mixed with 0.4 μ1 E.coli Poly(A) Polymerase (5U/μL), 2 μ1 10 × Poly(A) Polymerase Buffer, 4 μ1 5 × rATP solution and made up to 20 μl with RNase-Free ddH_2_O. The reaction was carried out at 37 °C for 60 min. For miRNA, 4 μl Poly (A) was mixed with 4 μl 10 × RT Primer (10 μM), 4 μl 10 × RT Buffer, 2 μl Super Pure dNTP (2.5 mM each), 2 μl RNase (40 U/μl), 1 μl Quant RTase and made up to 40 μl with 23 μl RNase-Free ddH_2_O. Reverse transcription was performed at 37 °C for 60 min. The resulting cDNA was stored in −80 °C until use.

Urinary levels of miRNAs were quantified by RT-QPCR using the ABI Prism® 7900 Sequence Detection System (Applied Biosystems, Foster City, CA, USA). For RT-QPCR, 10 μl 2 × miRcute miRNA Premix, 0.4 μl Forward Primer, 0.4 μl Reverse Primer (10 μM), 2 μl miRNA cDNA and 7.2 μl ddH_2_O were mixed to make a 20 μl reaction volume. Each sample was run in triplicate. Real-time quantitative polymerase chain reaction (RT-QPCR) was performed at 94 °C for 2 min, followed by 40 cycles at 94 °C for 20 s and 60 °C for 34 s. Results were analyzed with Sequence Detection Software, Version 2.0 (Applied Biosystems). RNA U6 was used as an endogenous control for normalizing the miRNA expression in urine sediments. The relative quantitation of miRNAs in the samples was evaluated as fold change calculated by the comparative Ct method (2^-△△Ct^).

### Evaluation of renal histopathological damage

The severity of histopathological damage was scored by two experienced pathologists who were blinded to the patients’ data, using the Oxford classification scoring system. The mesangial hypercellularity score was recorded as M1 for instances in which more than half the glomeruli have more than three cells in a mesangial area. Otherwise, the score was reported as M0. Endocapillary hypercellularity and segmental glomerulosclerosis were scored as present or absent (i.e. E1 or E0, and S1 or S0). Tubular atrophy/interstitial fibrosis was classified according to the percentage of cortical area involved by the tubular atrophy or interstitial fibrosis: <25% as T0, 26–50% as T1, and >50% as T2.

### Statistical analysis

Normally distributed data were expressed as mean ± SD and were compared using the *t*-test or one-way ANOVA. Nonparametric variables were expressed as medians, with corresponding 25th and 75th (interquartile range), and compared using the Mann–Whitney or Kruskal–Wallis tests. Categorical data were expressed as percentages and compared using the chi-squared test. Pearson or Spearman’s correlation was used to analyze correlations. We constructed receiver operating characteristic (ROC) curves and calculated the area under the ROC curve (AUC) to investigate the value of the miRNAs for predicting IgAN and evaluating histological injury. Statistical significance was considered as *P* < 0.05.

## Results

### Clinical and pathological characteristics of patients with IgAN

Fifty-two IgAN patients were enrolled in our study. Of them, 31 were males and 21 were females. 20 were treated with renin angiotensin system (RAS) blockade alone and 32 were treated with steroids and/or immunosuppressants. The age at renal biopsy was 33.45 ± 10.36 years old. The baseline characteristics of IgA nephropathy patients and healthy controls are listed in Table 1.

**Table 1:** The baseline characteristics of IgA nephropathy patients and healthy controls

	IgAN	Healthy Control
No. of cases	52	25
Sex (M/F)	31/21	13/12
Age (year)	33.45 ± 10.36	33.72 ± 7.77
Scr (μmol/L)	104.78 ± 46.30	60.04 ± 9.37
U-Prot (g/24 h)	1.99 (0.64–2.91)	0.029 (0.007–0.064)
eGFR (ml/mim/1.73 m^2^)	84.67 ± 32.45	131.25 ± 11.68
Oxford score		
M0/M1 (*n*)	31/21	–
E0/E1 (*n*)	47/5	–
S0/S1 (*n*)	28/24	–
T0/T1/T2 (*n*)	29/12/11	–

Note: Scr, serum creatinine; U-Prot, 24 h urinary protein excretion; eGFR, estimated glomerular filtration rate

### Levels of urinary miRNAs in IgAN patients and controls

**Fig. 1:**
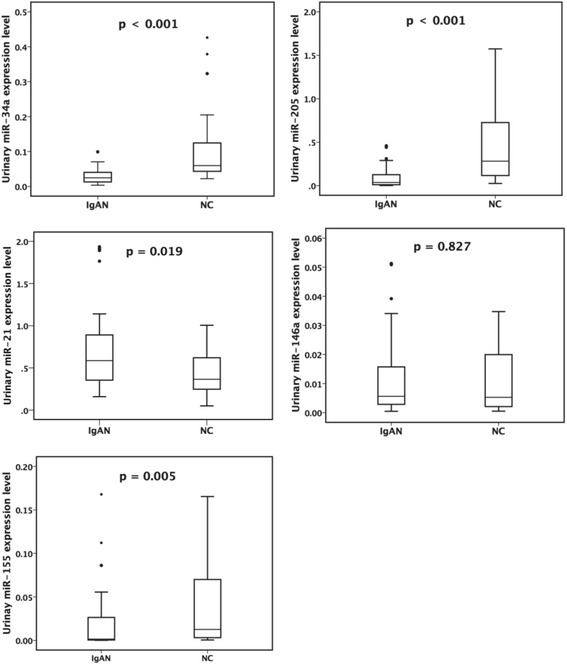
Urinary expression of miRNAs between patients with IgA nephropathy (IgAN) and normal controls (NC)

As shown in Fig. 1, the urinary expressions of miR-34a, miR-205, and miR-155 were significantly lower, while miR-21 were higher in the IgA group than in the NC group (0.025 (0.004–0.099) vs 0.060 (0.022–0.426), *p* < 0.001; 0.036 (0.002–0.459) vs 0.281 (0.025–1.573), *p* < 0.001; 0.001 (0.000–0.168) vs 0.013 (0.000–0.165), *p* = 0.005; 0.587 (0.159–1.936) vs 0.365 (0.050–1.005), *p* = 0.019). The levels of miR-146a were of no difference between two groups (0.006 (0.000–0.051) vs 0.005 (0.000–0.035), *p* = 0.827).

### Correlations between urinary miRNAs and clinical parameters

The levels of urinary miR-205 were positively correlated with eGFR (*r* = 0.316, *p* = 0.025), and negatively correlated with 24 h urinary protein excretion (U-Prot), cystatin, and uric acid (*r* = −0.415, *p* = 0.003; *r* = −0.500, *p* = 0.001; *r* = −0.389, *p* = 0.006). Expressions of miR-21 were positively correlated with U-Prot, cystatin (*r* = 0.362, *p* = 0.008; *r* = 0.462, *p* = 0.002), and negatively correlated with eGFR and urine osmosis (*r* = −0.481, *p* < 0.001; *r* = −0.385, *p* = 0.017).

### Correlations between urinary miRNAs and histopathological parameters

**Fig. 2:**
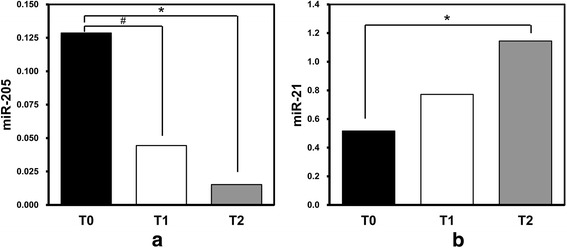
Correlations between urinary miRNAs and severity of tubular atrophy/interstitial fibrosis. **a**. Levels of urinary miR-205 in patients with different scores of tubular atrophy/interstitial fibrosis. **b**. Levels of urinary miR-21 in patients with different scores of tubular atrophy/interstitial fibrosis. #, significant difference between patients with T1 and T0. *, significant difference between patients with T2 and T0

For mesangial proliferation, the levels of urinary miR-205 were significantly lower in patients with M1 than those in patients with M0 (0.049 ± 0.070 vs 0.112 ± 0.129, *p* = 0.032). For segmental glomerulosclerosis and endocapillary hypercellularity, the levels of urinary miR-34a, miR-205, miR-21, miR-146a, and miR-155 showed no significant difference between patients with S1 and S0, E1 and E0. The levels of miR-205 decreased as the severity of tubular atrophy or interstitial fibrosis increased; they were significantly lower in patients with a score of T1 and T2 than those with T0 (0.044 ± 0.040 vs 0.129 ± 0.134, *p* = 0.011; 0.015 ± 0.011 vs 0.129 ± 0.134, *p* < 0.001) (Fig. 2a). However, the levels of miR-21 were upregulated in patients with severe tubular atrophy or interstitial fibrosis. They were higher in patients scored as T2 than in those scored as T0 (1.144 ± 0.656 vs 0.516 ± 0.283, *p* = 0.036) (Fig. 2b). Besides, crescents existed in 27 (51.9%) biopsy specimens of 52 IgAN patients. The levels of miR-21 were positively correlated with the ratios of crescents (*r* = 0.294, *p* = 0.035). The association between the urinary miRNAs and the clinical and pathological characteristics were summarized in a synthetic table (Additional file 1).

### Prediction values of urinary miRNAs in IgAN patients

**Fig. 3:**
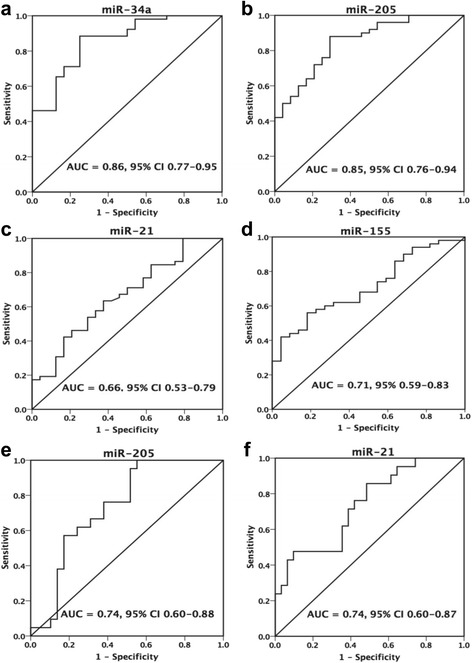
Receiver operating characteristic (ROC) curve analysis of the prediction values of miRNAs in IgAN patients. **a**-**d** ROC curve of discriminating IgAN from healthy controls. **e**-**f** ROC curve of discriminating IgAN patients with tubular atrophy/interstitial fibrosis T1 and T2 from patients with T0. 95% CI, 95% confidence interval; AUC, area under the curve

ROC analysis revealed that urinary miR-34a, miR-205, miR-21, and miR-155 levels discriminated patients with IgAN from NC, with AUC values of 0.86, 0.85, 0.66 and 0.71, respectively. The cutoff values were miR-34a ≤ 0.047 (Fig. 3a), miR-205 ≤ 0.209 (Fig. 3b), miR-21 ≥ 0.461 (Fig. 3c), and miR-155 ≤ 0.002 (Fig. 3d). More importantly, miR-205 and miR-21 can distinguish patients with T1 and T2 tubular atrophy/interstitial fibrosis from patients with T0, with AUC values of 0.74 and 0.74. The cutoff values were miR-205 ≤ 0.125 (Fig. 3e) and miR-21 ≥ 0.891 (Fig. 3f).

### Correlation between urinary miRNAs and clinical remission

Of the 52 IgAN patients, one provided only routine urine protein tests, such as 2+, but no exact 24 h urine proteinuria. The mean time of follow-up was 15.19 months (10–19.67 months). There were no correlations between the rate of eGFR loss (ml/min/1.73 m^2^/year) and levels of baseline miRNAs. The reduction of U-prot (g/24 h/year) were correlated positively with baseline miR-21 (*r* = 0.288, *p* = 0.041), and correlated negatively with miR-205 (*r* = −0.414, *p* = 0.003). We divided the 51 patients into CR and non-CR groups after the follow-up. The results are summarized in Table 2. The levels of baseline miR-205 in the CR group were significantly higher than non-CR group (*p* = 0.018), while miR-21 and miR-146a were lower than non-CR group (*p* = 0.021; *p* = 0.009). The patients who achieved a complete remission of proteinuria also had severer tubular atrophy/interstitial fibrosis (*p* = 0.005). We further investigated the factors associated with CR using logistic analysis. Only higher baseline eGFR contributed to the CR in IgAN patients (*p* = 0.001, OR = 1.042). Considering the effect of different therapies, we investigated the correlation of baseline parameters and remission of proteinuria under different therapies. In patients treated with steroids and/or immunosuppressants, the levels of baseline miR-146a in the CR group were significantly lower than non-CR group (0.004 (0.001–0.006) vs 0.010 (0.004–0.023), *p* = 0.014). While in patients treated with RAS blockers alone, the levels of baseline miR-205 in the CR group were significantly higher than non-CR group (0.139 (0.076–0.295) vs 0.012 (0.009–0.061), *p* = 0.021).

**Table 2:** Comparison of baseline clinical parameters and urinary miRNAs levels between CR and non-CR groups

	CR	Non-CR	*P* value
No. of patients, *n* (%)	27	24	
Age (years)	32.52 ± 10.66	33.83 ± 9.81	0.650
Baseline GFR (ml∙min^−1^1.73 m^−2^)	100.91 ± 23.12	66.90 ± 32.94	<0.001*
Baseline proteinuria (g/d)	0.77 (0.62–1.58)	2.55 (1.03–4.47)	0.002*
Baseline ALB (g/L)	41.08 ± 4.19	36.13 ± 8.88	0.018*
Baseline NAG	32.74 ± 22.57	44.30 ± 35.99	0.218
Baseline osmosis	713.40 ± 218.80	592.22 ± 187.28	0.080
miR-34a	0.031 ± 0.023	0.026 ± 0.018	0.440
miR-205	0.113 ± 0.123	0.045 ± 0.058	0.018*
miR-21	0.537 ± 0.301	0.857 ± 0.584	0.021*
miR-146a	0.003 (0.002–0.010)	0.009 (0.004–0.023)	0.009*
miR-155	0.002 (0.000–0.028)	0.002 (0.001–0.030)	0.298
M0/M1	20/7	12/12	0.076
E0/E1	26/1	24/0	1.000
S0/S1	16/11	13/11	0.714
T0/T1/T2	20/6/1	10/5/9	0.005*

*, Statistical significance with *P* < 0.05

## Discussion

The clinical course of IgAN is extremely variable, ranging from minor urinary abnormalities to rapidly progressive renal failure. Thus, the investigation of noninvasive and more reliable biomarkers to sequentially assess kidney injury and disease progression is crucial. Recent studies found that miRNAs can be secreted by cells into body fluids and these circulating miRNAs are highly stable [26–30]. Produced by the kidney and passing through almost all kidney cell types, urine may contain miRNAs that can serve as biomarkers for kidney diseases [31, 32].

MiR-34a induces G1 arrest and inhibits cell proliferation by regulation of several cell cycle genes, including cyclin D1 (CCND1), cyclin E2 (CCNE2), CDK4, CDK6 [33, 34]. MiR-34a is also reported to regulate cell proliferation by reducing the levels of anti-apoptotic protein BCL-2 [35] and transcription factor E2F3 [36]. Our studies showed that urinary expressions of miR-34a were significantly lower in IgAN than in NC group, but there were no significant correlations between miR-34a levels, clinical indicators, and pathological damages in IgAN. Studies with larger sample and basic experiments are needed to confirm the roles of miR-34 in the pathogenesis of IgAN.

Renal fibrosis is the final outcome of many chronic kidney diseases [37]. Activated myofibroblasts and epithelial-mesenchymal transition (EMT) play important roles in the progression of renal fibrosis [38]. It is reported that tubulointerstitial fibrosis is an important factor affecting the development and prognosis of IgAN [39, 40]. One of the hallmarks of EMT is the loss of E-cadherin, which responsible for cell-cell adhesion and maintenance of cytoskeletal organization. Transcription factors ZEB1 and ZEB2 can inhibit the transcription of E-cadherin [11]. Studies have shown that miR-205 might mediate the repression of ZEB1 and ZEB2, resulting in E-cadherin up-regulation and EMT repression [10]. There is increasing evidence that miR-21, which promotes fibrosis, plays a major role in the progression of renal disease [41, 42]. Glowacki F et al. [43] found miR-21 to be highly upregulated in the kidneys of mice with unilateral ureteral obstruction (UUO) and in the kidneys of patients with severe kidney fibrosis. Zhong X et al. [44] also found that miR-21 participates in renal fibrosis through positively regulating the expression of extracellular matrix (ECM) and a-SMA in tubular epithelial cells (TECs) and fibrotic kidneys. Our study showed that the IgAN group had significantly lower urinary miR-205 but higher miR-21 levels than controls. The ROC revealed that miR-205 ≤ 0.125 and miR-21 ≥ 0.891 can distinguish patients with severe tubular atrophy/interstitial fibrosis from patients with mild tubular atrophy/interstitial fibrosis. These data indicate that the urinary miR-205 and miR-21 parallel with the severity of tubulointerstitial damage in IgAN.

Our results showed no correlations between miR-146a, miR-155 and clinical parameters and histological features. There is a negative feedback loop in immune cells. Stimulation of TNF receptor associated factor 6 (TRAF6) could enhance NF-κB activity, which upregulates miR-146a expression. The increased miR-146a level, in turn, suppresses TRAF6 and NF-kB activity, which properly terminates the immune response [45]. The urinary samples of IgAN that were collected in different periods of the negative feedback may lead to controversial results.

Proteinuria is a strong prognostic factor for IgAN progression [46, 47]. Reich et al. reported that persistent proteinuria is the strongest predictor of poor renal outcome in IgAN and that sustained reduction of proteinuria to <1 g/24 h is associated with a good prognosis [48]. In this study, we regarded the remission of proteinuria as main clinical outcome. The results showed that the reduction of U-Prot (g/24 h/month) was correlated with baseline urinary miR-21 and inversely correlated with miR-205. The subjects who achieved a complete remission had higher baseline urinary miR-205, lower miR-21 and miR-146a than those did not achieve a complete remission. Altogether, these data suggest miR-205 and miR-21 might be novel, reliable urinary biomarkers of kidney fibrosis and predictors of progression of IgAN. Targeting miR-205 and miR-21 might provide significant therapeutic effects in IgAN.

Our study has limitations. First, we detected the urinary miRNA expressions in urine sediments, which consist of different cell types, such as red blood cells, lymphocytes, and renal tubular cells. We did not determine their cellular source. In addition, healthy people were used as controls in this study. The results were helpful to distinguish patients with IgAN from healthy people, but not exclude an association between other chronic kidney diseases and miRNAs. Including another cohort of patients with kidney disease other than IgAN will be more conclusive. This was a single-center study with a limited number of patients. Studies involving a larger sample and followed up for a longer time are needed in further.

## Conclusions

In conclusion, in the first part of the study, we showed a significant reduction of miR-34a, miR-205, and miR-155 but an increase of miR-21 in the urine of patients with IgAN. Then, we demonstrated a significant correlation between the urinary miR-205 and miR-21 with histologic lesions, especially tubulointerstitial damages. Finally, we assessed the prognosis effect of urinary miRNAs on remission of proteinuria. The levels of miR-205, miR-21 and miR-146a, measured at the time of renal biopsy, predicted the outcome in IgAN patients. These findings supported the notion that urinary miRNAs can serve to reflect tubulointerstitial damage and predict the progression of IgAN.

## Additional file

Additional file 1: Association between the urinary miRNAs and the clinical and pathological characteristics. (DOCX 102 kb)

## Abbreviations

ACEIs: Angiotensin-converting enzyme inhibitors
ARBs: Angiotensin receptor blockers
AUC: Area under the ROC curve
CCND1: Cyclin D1
CCNE2: Cyclin E2
CKD-EPI: Chronic kidney disease epidemiology
CR: Complete remission
DEPC: Diethylpyrocarbonate
ECM: Extracellular matrix
EMT: Epithelial-mesenchymal transition
ESRD: End-stage renal disease
GFR: Glomerular filtration rate
H & E: Hematoxylin and eosin
IgAN: IgA nephropathy
KDIGO: Kidney Disease: Improving Global Outcomes
miRNAs: MicroRNAs
NC: Normal control
NR: No response
PAS: Periodic acid–Schiff stain
PASM: Periodic acid silver methenamine
PR: Partial remission
RA: Rheumatoid arthritis
RAS: Renin angiotensin system
ROC: Receiver operating characteristic
RT-QPCR: Real-time quantitative polymerase chain reaction
TECs: Tubular epithelial cells
TGF-β: Transforming growth factor beta
TLR: Toll-like receptor
TRAF6: TNF receptor associated factor 6
U-Prot: 24 h urinary protein excretion
UUO: Unilateral ureteral obstruction
