# Supplementary material for: Urinary sediment miRNAs reflect tubulointerstitial damage and therapeutic response in IgA nephropathy
Source: BMC Nephrol. 2017 Feb 15;18:63. doi: 10.1186/s12882-017-0482-0 (PMC5312444; doi:10.1186/s12882-017-0482-0)
Supplement: Additional file 1: — Association between the urinary miRNAs and the clinical and pathological characteristics. (DOCX 102 kb) [file 12882_2017_482_MOESM1_ESM.docx]

**Additional file 1. Association between the urinary miRNAs and the clinical and pathological characteristics**

|  | **eGFR** | **U-Prot** | **cystatin** | **uric acid** | **urine osmosis** | **crescents** |
| --- | --- | --- | --- | --- | --- | --- |
| miR-34a | r = -0.042，  p = 0.770 | r = 0.017，  p = 0.905 | r = -0.090，  p = 0.566 | r = -0.187，  p = 0.194 | r = 0.034，  p = 0.839 | r = -0.005，  p = 0.970 |
| miR-205 | r = 0.316，  p = 0.025 | r = -0.415，  p = 0.003 | r = -0.500，  p = 0.001 | r = -0.389，  p = 0.006 | r = -0.016，  p = 0.928 | r = -0.107，  p = 0.458 |
| miR-21 | r = -0.481，  p＜0.001 | r = 0.362，  p = 0.008 | r = 0.462,  p = 0.002 | r = 0.029，  p = 0.844 | r = -0.385，  p = 0.017 | r = 0.294，  p = 0.035 |
| miR-146a | r = -0.206，  p = 0.143 | r = 0.136，  p = 0.338 | r = 0.348，  p = 0.022 | r = 0.083，  p = 0.566 | r = -0.186，  p = 0.263 | r = -0.175，  p = 0.215 |
| miR-155 | r = -0.077，  p = 0.595 | r = 0.158，  p = 0.272 | r = 0.214，  p = 0.180 | r = -0.144，  p = 0.330 | r = -0.269，  p = 0.107 | r = 0.056，  p = 0.702 |

|  | **miR-34a** | **P**  **value** | **miR-205** | **P**  **value** | **miR-21** | **P value** | **miR-146a** | **P value** | **miR-155** | **P value** |
| --- | --- | --- | --- | --- | --- | --- | --- | --- | --- | --- |
| **Mesangial hypercellularity score** | | | | | | | | | | |
| **M0** | 0.031±0.020 |  | 0.112±0.129 |  | 0.543（0.316-0.853） |  | 0.004（0.002-0.010） |  | 0.001（0.000-0.020） |  |
| **M1** | 0.025±0.020 | 0.288 | 0.049±0.070 | 0.032 | 0.661（0.356-0.913） | 0.334 | 0.007（0.004-0.023） | 0.098 | 0.002（0.001-0.032） | 0.494 |
| **Segmental glomerulosclerosis** | | | | | | | | | | |
| **S0** | 0.032±0.020 |  | 0.042（0.014-0.126） |  | 0.555（0.274-1.053） |  | 0.004（0.003-0.009） |  | 0.027±0.043 |  |
| **S1** | 0.025±0.021 | 0.249 | 0.027（0.009-0.161） | 0.492 | 0.602（0.357-0.725） | 0.834 | 0.009（0.003-0.020） | 0.216 | 0.010±0.017 | 0.088 |
| **Tubular atrophy/interstitial fibrosis** | | | | | | | | | | |
| **T0** | 0.027（0.012-0.042） |  | 0.129±0.134 |  | 0.516±0.283 |  | 0.004（0.003-0.010） |  | 0.001（0.000-0.031） |  |
| **T1** | 0.024（0.011-0.033） |  | 0.044±0.040 | 0.011^#^ | 0.772±0.464 |  | 0.004（0.002-0.023） |  | 0.002（0.000-0.017） |  |
| **T2** | 0.023（0.015-0.029） | 0.880 | 0.015±0.011 | < 0.001^*^ | 1.144±0.656 | 0.036^*^ | 0.013（0.005-0.022） | 0.178 | 0.001（0.001-0.015） | 0.926 |

#, significant difference between patients with T1 and T0

*, significant difference between patients with T2 and T0
